# Effects of intensity and amount of exercise on measures of insulin and glucose: Analysis of inter-individual variability

**DOI:** 10.1371/journal.pone.0177095

**Published:** 2017-05-11

**Authors:** Louise de Lannoy, John Clarke, Paula J. Stotz, Robert Ross

**Affiliations:** 1 School of Kinesiology and Health Studies, Queen’s University, Kingston, Ontario, Canada; 2 School of Medicine, Division of Endocrinology and Metabolism, Queen’s University, Kingston, Ontario, Canada; Florida International University Herbert Wertheim College of Medicine, UNITED STATES

## Abstract

**Aim:**

To determine the separate effects of exercise amount and intensity on the rate of response for glucose and insulin variables, where rate of response was defined as the number of individuals with improvement in glucose and insulin values that was beyond the day-to-day variability of measurement.

**Methods:**

Participants were 171 sedentary, middle-aged abdominally obese adults who completed a 24-week intervention. Participants were randomly assigned to (1) no-exercise control (n = 51), (2) low-amount, low-intensity exercise (LALI, n = 38), (3) high-amount, low-intensity exercise (HALI, n = 52), or (4) high-amount, high-intensity exercise (HAHI, n = 30). Two-hour glucose, insulin area under the curve (AUC), and fasting insulin were measured during a 2-hour, 75g oral glucose challenge. The day-to-day variability for these measures was calculated to be ±2.2 mmol/L, ±940.2 pmol/L, and ±38.9 pmol/L, respectively.

**Results:**

At 24 weeks, the number of nonresponders for 2-hr glucose was 98.0%, 86.8%, 94.2%, 86.7% in the control, LALI, HALI, and HAHI groups, respectively. The number of nonresponders for insulin AUC was 88.0%, 75.7%, 75.0%, 80.0% in the control, LALI, HALI, and HAHI groups, respectively. The number of nonresponders for fasting insulin was 88.2%, 84.2%, 84.6%, 93.3% in the control, LALI, HALI, and HAHI groups, respectively. The rate of response was not different between control and any of the exercise groups for any measure (p>0.05).

**Conclusion:**

The improvement in glucose and insulin measures did not exceed the day-to-day variability of measurement for approximately 80% of the participants independent of exercise amount or intensity.

## Introduction

Heritability estimates for baseline fasting glucose range from 10 to 75% and fasting insulin from 20 to 55% [1]. This range of variability for not only intrinsic, but also acquired characteristics, will doubtless influence the individual response to a standard exercise dose. Despite this recognition individual variability in glucose and insulin response to exercise is a largely neglected phenomenon, with few exceptions [2, 3]. This represents a missed opportunity as characterizing the determinants that contribute to individual variability in response to exercise can be a first step towards greater precision in individual, lifestyle-based treatments.

When considering the individual response to treatment, it is important to interpret the response in concert with the day-to-day variability in measurement. This variability consists of both biological variability–variability due to fluctuations in free-living habits such as dietary composition [4], sleep patterns [5], and stress level [6]–as well as analytical variability. The biological variability for insulin measures has been documented at upwards of 50–60% [4, 7]. We [8, 9] and others [2, 10] argue that a true treatment benefit for a given individual would be a response that exceeds the random day-to-day variability of measurement. Consideration of whether an exercise regimen has effects on glucose and insulin metabolism that is beyond the biological and analytical variability, as measured by technical error, is another neglected aspect of intervention trials. While Bouchard and colleagues [2] recently demonstrated the large range of response in metabolic measures, above and below the technical error of measurement following an exercise regimen, it is unknown whether alterations in the amount and/or intensity of exercise influences the number of participants whose response is beyond the day-to-day variability of measurement for measures of insulin and glucose.

In this study we sought to determine the individual and combined effects of amount and intensity of exercise on the individual response in measures of glucose and insulin, where the rate of response is defined as the number of individuals whose improvement in glucose and insulin was beyond the day-to-day variability. We also considered whether exposure to exercise and/or exercise adherence influenced the rate of response.

## Methods

### Study setting and participants

Details of the trial design [11] and the primary findings [12] have been published (ClinicalTrials.gov: NCT00955071). We conducted a 24-week, single-center, randomized controlled trial with a parallel group design between September 1, 2009, and May 31, 2013. The primary objective of the original investigation was to determine the separate effects of exercise intensity and amount on waist circumference and glucose tolerance among 300 sedentary, abdominally obese adults. Potential participants were excluded if they reported a history of heart disease, stroke, or any condition that would prevent them from engaging in exercise, if they were already engaged in 2 or more planned exercise sessions per week, and if they had diabetes. All participants provided written informed consent before participation, and the study was originally approved by the Queen’s University Health Sciences Research Ethics Board (Approval code: PHE-093-09).

The purpose of the current analysis was to examine the separate effects of exercise intensity and amount on the individual variability of response for measures of insulin and glucose. Of the 300 participants originally randomized, participants were excluded from the final data set for this secondary analysis if they did not complete the study and/or did not have follow-up 2-hour glucose data, and/or insulin area under the curve (AUC) data, and/or fasting insulin data (n = 84 for 2-hour glucose, n = 86 for insulin AUC, n = 84 for fasting insulin), had a baseline 2-hour glucose level below the physiologically normal range (<4mmol/L; n = 5) or had an exercise adherence (number of exercise sessions attended) of less than 90% (n = 40). This resulted in a study sample of 171 participants.

### Exercise intervention

Participants were randomly assigned to (1) no-exercise control (n = 51), (2) low-amount, low-intensity exercise (LALI; n = 38), (3) high-amount, low-intensity exercise (HALI; n = 52), or (4) high-amount, high-intensity exercise (HAHI, n = 30). All participants in the exercise groups performed primarily walking exercise on a treadmill for the time required to achieve the desired energy expenditure (kcal per session) 5 times per week at the required intensity (relative to cardiorespiratory fitness (VO2peak)) for 24 weeks. Using the heart rate and oxygen consumption data obtained from the baseline fitness (VO2peak) test, the heart rate associated with an oxygen consumption of approximately 50% (LALI and HALI) and approximately 75% (HAHI) were prescribed for each participant. At these exercise intensities, the energy expenditure targets (exercise amount) for women and men were 180 and 300 kcals, respectively, for LALI and 360 and 600 kcals for both HALI and HAHI. These exercise doses are based on previous calculations of VO_2peak_ in sedentary, abdominally obese adults [13, 14]. Energy expenditure targets for the low amount group were prescribed so that this energy would be expended in approximately 30 minutes to conform to physical activity guidelines [11, 15]. Energy expenditure targets for the high amount group (matched for intensity) was designed such that this energy would be expended in approximately twice the amount of time, 60 minutes, as the low-amount group [11]. Prescribing dose based on energy expenditure rather than time was done to reduce inter-individual variability in exercise capacity [11]. Heart rate was monitored continuously for all exercise participants every session to help ensure adherence to the prescribed exercise intensity. All exercise sessions were performed under supervision.

### Accelerometry

Physical activity performed outside of the supervised exercise sessions (daily physical activity) was monitored using ActiGraph GT3X accelerometers for a 1-week period at baseline, 16 and 25 weeks. Participants wore the accelerometer for at least 4 days, 10 hours/day each period. Established accelerometer cut points were used to estimate the duration and intensity of physical activity and sedentary behavior [16].

### Dietary regimen

During a 1-week baseline period, participants were instructed to maintain baseline body weight through maintenance of caloric intake while recording their daily consumption of self-selected foods. During the intervention, participants were instructed to maintain the caloric intake targets determined during baseline. All participants were prescribed a balanced diet and were asked to submit daily diet intake records for the duration of the intervention [11].

### Insulin and glucose measures

Two-hour glucose level and fasting insulin were measured during a 2-hour, 75g oral glucose tolerance test (OGTT) between 36 and 48 hours after the last exercise session at baseline, 16 and 24 weeks. Insulin AUC values were obtained at 24 weeks. Waist circumference was measured at the superior edge of the iliac crest. Weight was measured using the same calibrated beam scale throughout the trial. Fasting glucose and insulin were measured using established procedures [11]. There were separate assessment and intervention personnel, and all assessment personnel were blinded to participant randomization assignment.

### Determination of day-to-day variability of measurement

Day-to-day variability, or technical error (TE) of measurement, was used to identify nonresponders for 2-hour glucose, insulin AUC, and fasting insulin based on methods outlined by Bouchard and colleagues [2] and as originally used in the National Health and Nutrition Examination Survey [17]. Briefly, the TE is calculated by taking the square root of the sum of squared differences of repeat measures divided by the total number of *n* paired samples multiplied by 2:

$$TE=\sqrt{\frac{\sum {\left(measure1-measure\;2\right)}^{2}}{2n}}$$

Our repeat measure data for glucose and insulin measures was obtained using the control group data at baseline and 24 weeks (N = 51). Our derived TE values for 2-hr glucose and insulin AUC are similar to values reported in the literature [2, 4, 18, 19]. Any value within 2 TEs was considered a nonresponse. Accordingly, the threshold values to determine nonresponse were calculated to be within ±2.2 mmol/L for 2-hour glucose, ±940.2 pmol/L for insulin AUC, and ±38.9 pmol/L for fasting insulin.

### Statistical analysis

A one-way analysis of variance was performed to compare variables across groups at baseline. Least significant difference post hoc tests were performed to further identify significance when necessary. McNemar’s tests were performed to compare the rate of response between time points 16 and 24 weeks for each group. The Marascuillo procedure was performed to compare the rate of response between groups. A Mann-Whitney U test was performed to compare change in total physical activity, sedentary time, and weight at 24 weeks between responders and nonresponders within all groups. Linear regression was performed to determine whether change in total physical activity, sedentary time, or weight predicted change in 2-hour glucose, insulin AUC, and fasting insulin at 24 weeks. Adjusted Wald confidence intervals (95%) were calculated for rate of response using the binary rate of response per group to calculate the mean and standard error [20]. Statistical significance was set at p<0.05. All statistical analyses were performed using SPSS software, version 23.0 (SPSS Inc).

## Results

### Baseline characteristics

Participant characteristics are summarized in Table 1. There were no significant differences in baseline characteristics between groups (p>0.05).

**Table 1:** Baseline characteristics of the 171 study participants.

	LALI (n = 38)	HALI (n = 52)	HAHI (n = 30)	Control (n = 51)
**Age, y**	53.7 (7.1)	52.6 (8.1)	53.8 (7.4)	52.5 (8.3)
**Waist circumference, cm**	109.9 (11.1)	110.5 (11.8)	111.3 (12.0)	109.1 (10.7)
**Body Mass Index, kg/m**^**2**^	33.0 (3.9)	33.3 (5.2)	33.0 (3.8)	32.9 (4.7)
**Weight, kg**	92.7 (13.9)	95.4 (19.2)	96.5 (16.2)	94.1 (17.0)
**Fasting Glucose, mmol/L**	5.4 (0.5)	5.4 (0.5)	5.5 (0.4)	5.5 (0.5)
**2-hr glucose, mmol/L**	7.5 (1.1)	7.3 (1.6)	7.2 (1.5)	7.7 (1.6)
**Fasting Insulin, pmol/L**	69.0 (40.1)	64.4 (40.2)	66.1 (32.8)	71.9 (35.0)
**Insulin AUC, pmol/L**	2357.5 (1397.9)	1995.5 (1339.9)	2016.5 (1175.2)	2082.0 (1015.9)
**Homeostatic model assessment–insulin resistance**	2.5 (1.6)	2.3 (1.5)	2.4 (1.2)	2.6 (1.3)
Cardiorespiratory fitness, L*min^**-1**^	2.6 (0.7)	2.8 (0.7)	2.7 (0.7)	2.8 (0.8)
**Sedentary Time (min/d)**	644.6 (91.0)	629.7 (81.3)	619.8 (84.3)	617.2 (89.0)
**Total Physical Activity (min/d)**	305.0 (78.3)	311.9 (76.1)	300.9 (95.3)	304.8 (90.8)

Values are means (standard deviations), unless otherwise noted.

### Variability of response for 2-hour glucose

**Fig 1:**
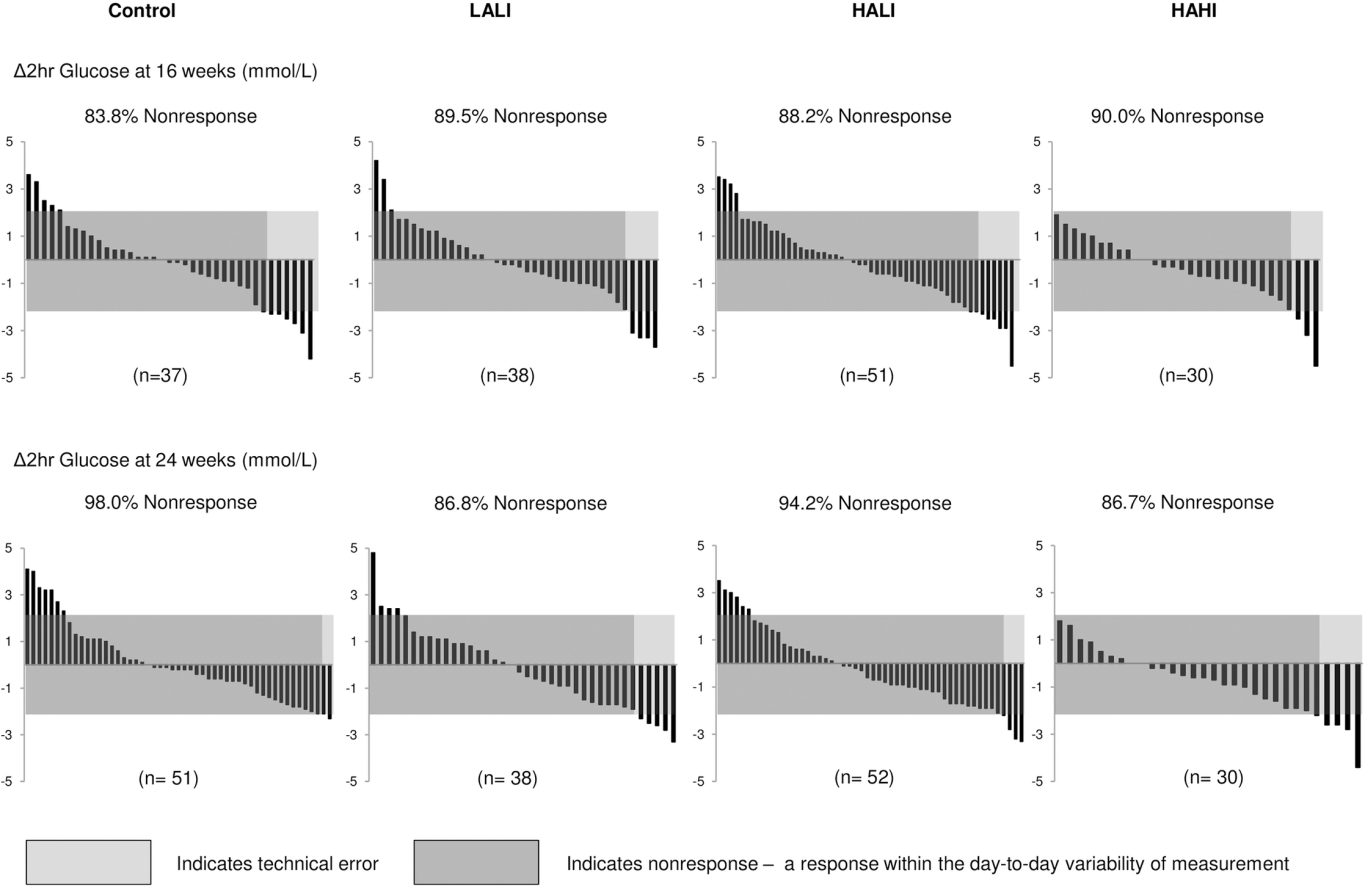
Change in 2-hour glucose for each participant at 16 and 24 weeks across groups. LALI = low amount, low intensity exercise; HALI = high amount, low intensity exercise; HAHI = high amount, high intensity exercise. 2TEs = 2.2 mmol/L.

Fig 1 illustrates the individual variability in 2-hour glucose response at 16 and 24 weeks. At 24 weeks, the number of nonresponders was 98.0%, 86.8%, 94.2%, and 86.7% in the control, LALI, HALI, and HAHI groups respectively, meaning that 2.0% (95% CI, -2.0 to 6.0%), 13.2% (CI, 2.0 to 20.0%), 5.8% (CI, -0.6 to 10.0%), and 13.3% (CI, 1.0 to 30.0%) improved 2-hour glucose, respectively, beyond the day-to-day variability in measurement. However, the number of nonresponders was not statistically different between control and any of the exercise groups (p>0.05). There was no significant improvement in the rate of response for 2-hour glucose between 16 and 24 weeks within all exercise groups (p>0.05).

### Variability of response for insulin AUC

**Fig 2:**
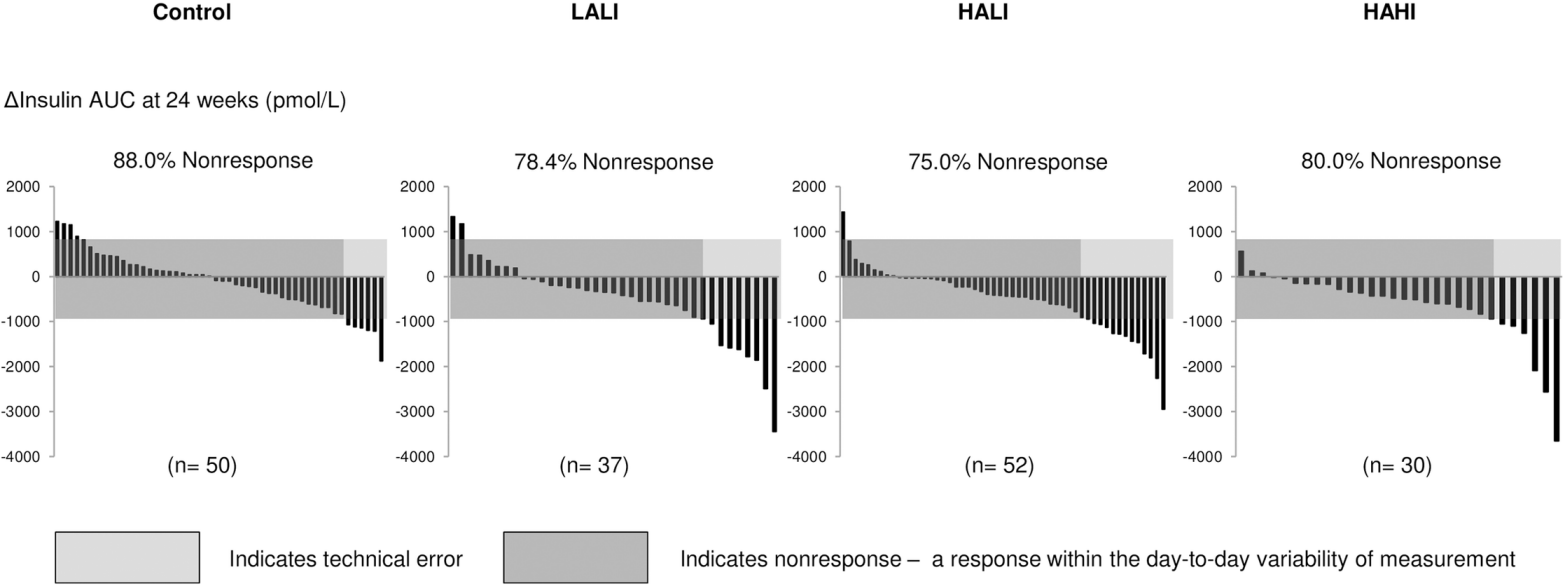
Change in insulin AUC for each participant at 24 weeks across groups. LALI = low amount, low intensity exercise; HALI = high amount, low intensity exercise; HAHI = high amount, high intensity exercise. 2TEs = 940.2 pmol/L.

Fig 2 illustrates the individual variability in insulin AUC response for all 4 groups at 24 weeks. At 24 weeks, the number of nonresponders was 88.0%, 75.7%, 75.0%, and 80.0% in the control, LALI, HALI, and HAHI groups, respectively, whereas 12.0% (CI, 3.0 to 20.0%), 24.3% (CI, 10.0 to 40.0%), 25% (CI, 10.0 to 40.0%), and 20.0% (CI, 6.0 to 30.0%) improved in insulin AUC beyond 2 TEs. The number of insulin AUC nonresponders was not different for any exercise group compared to control (p>0.05).

### Variability of response for fasting insulin

**Fig 3:**
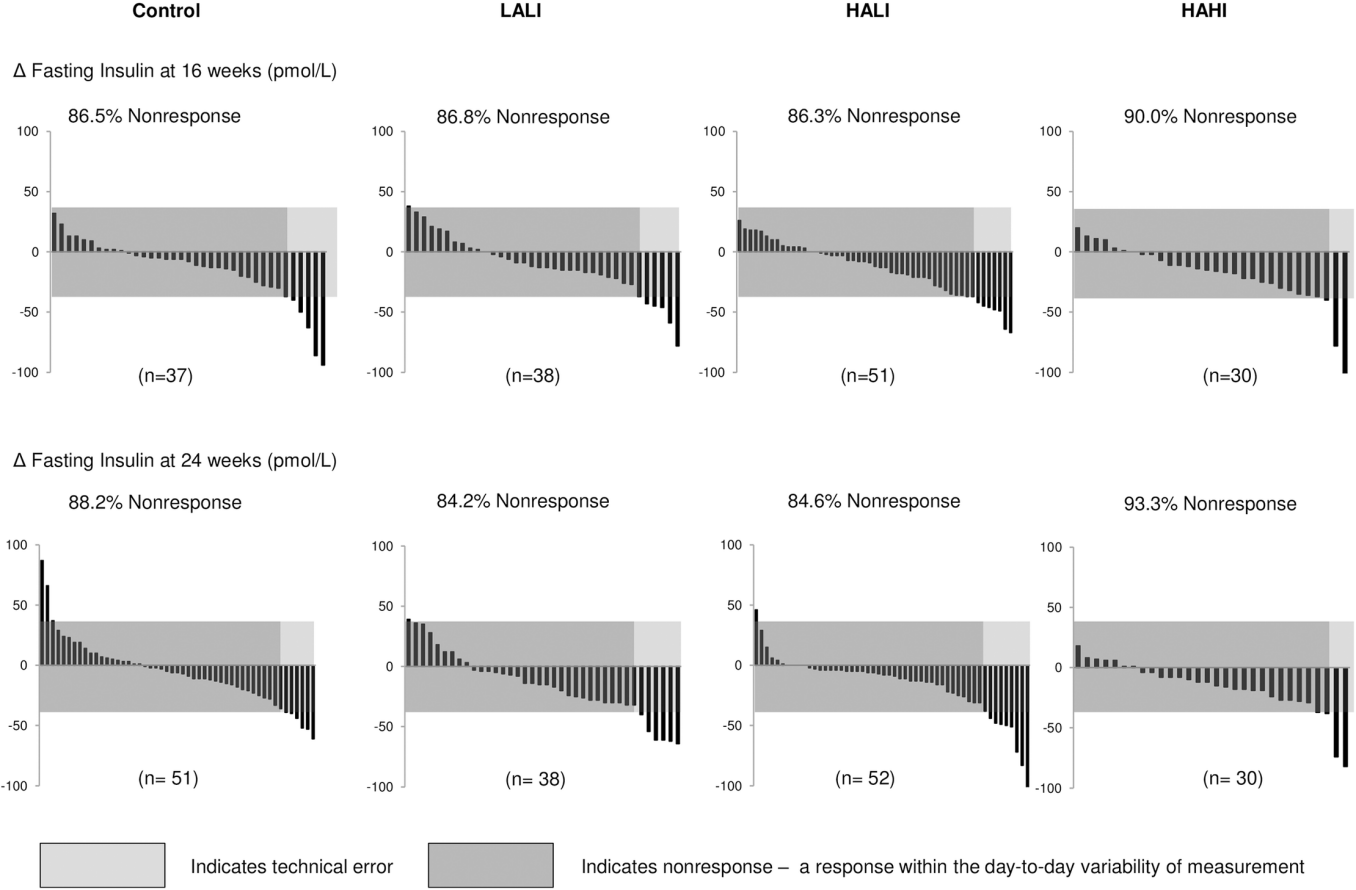
Change in fasting insulin for each participant at 24 weeks across groups. LALI = low amount, low intensity exercise; HALI = high amount, low intensity exercise; HAHI = high amount, high intensity exercise. 2TEs = 38.9 pmol/L.

Fig 3 illustrates the individual variability in fasting insulin response for all 4 groups at 16 and 24 weeks. At 24 weeks, the number of nonresponders was 88.2%, 84.2%, 84.6%, and 93.3% in the control, LALI, HALI, and HAHI groups, respectively, meaning that 11.8% (CI, 2.0 to 20.0%), 15.8% (CI, 4.0 to 30.0%), 15.4% (CI, 6.0 to 30.0%), and 6.7% (CI, -2.0 to 20.0%) improved in fasting insulin, respectively, beyond 2 TEs. As with insulin AUC, the number of nonresponders for fasting insulin was not different between control and any of the exercise groups (p>0.05). There was no significant improvement in the rate of response for fasting insulin between 16 and 24 weeks within all exercise groups (p>0.05).

### Variability of response for change in body weight with glucose and insulin measures

**Fig 4:**
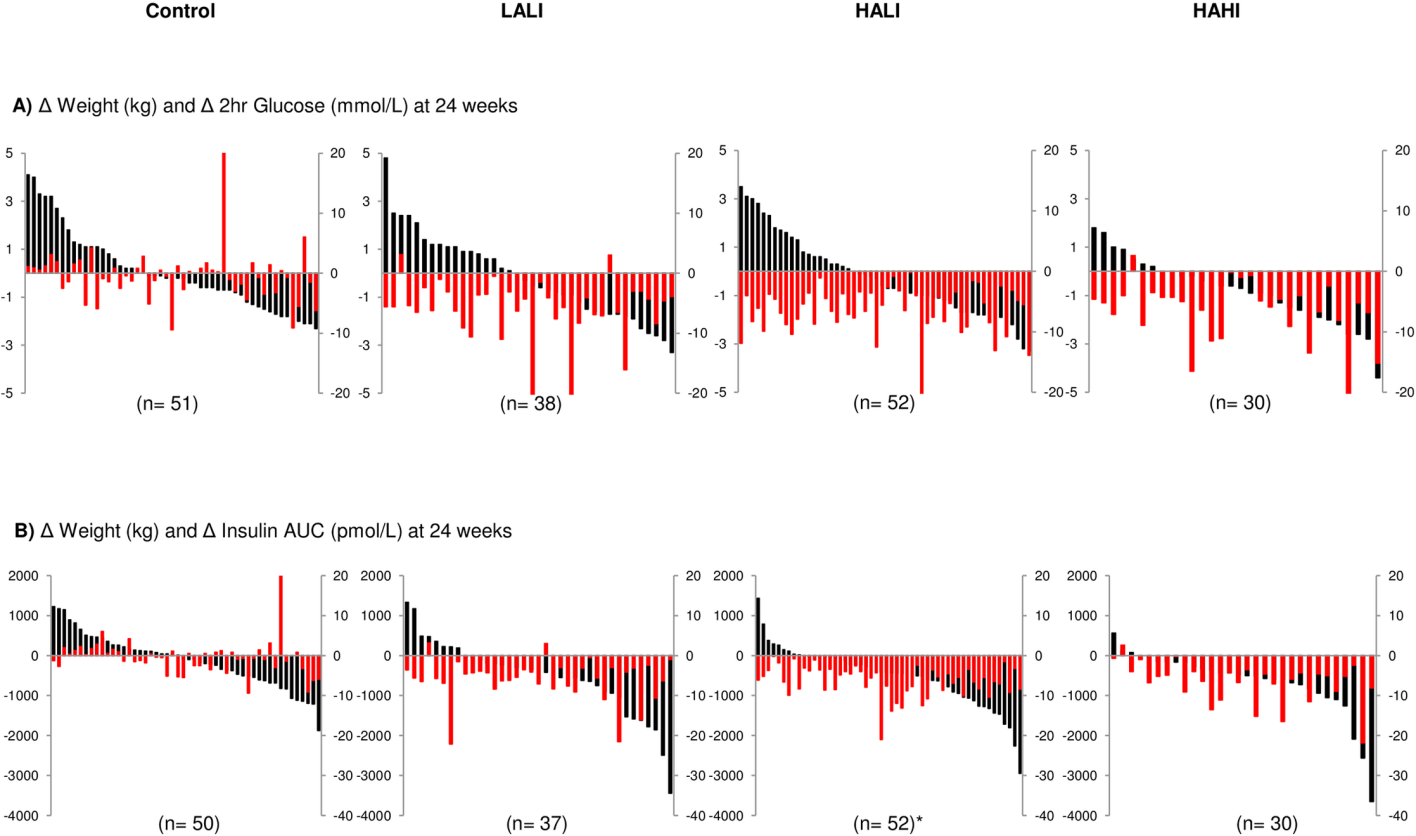
Change in body weight associated with 2-hour glucose and insulin AUC change for each participant across 24 weeks. (A) Weight change (red bars; kg) with 2-hour glucose change (black bars; mmol/L) and (B) Weight change (red bars; kg) with insulin AUC change (black bars; pmol/L). Data for fasting insulin not shown. LALI = low amount, low intensity exercise; HALI = high amount, low intensity exercise; HAHI = high amount, high intensity exercise. * indicates significant difference in weight change between responders and nonresponders at p<0.05.

The individual change scores for body weight with 2-hr glucose and insulin AUC are illustrated in Fig 4. With the exception of insulin AUC in HALI (p = 0.03), there were no differences in the body weight change observed between responders and nonresponders for any variable for all intervention groups (p>0.05). Change in body weight was not associated with change in any glucose or insulin variable for all intervention groups (p>0.05), however, change in body weight was significantly associated with change in insulin AUC within all intervention groups, though the variance explained was small (R^2^ = 0.18, p = 0.003; R^2^ = 0.05, p = 0.04; R^2^ = 0.07, p = 0.007 for LALI, HALI, and HAHI, respectively).

### Variability of response for daily physical activity and sedentary time with glucose and insulin measures

**Fig 5:**
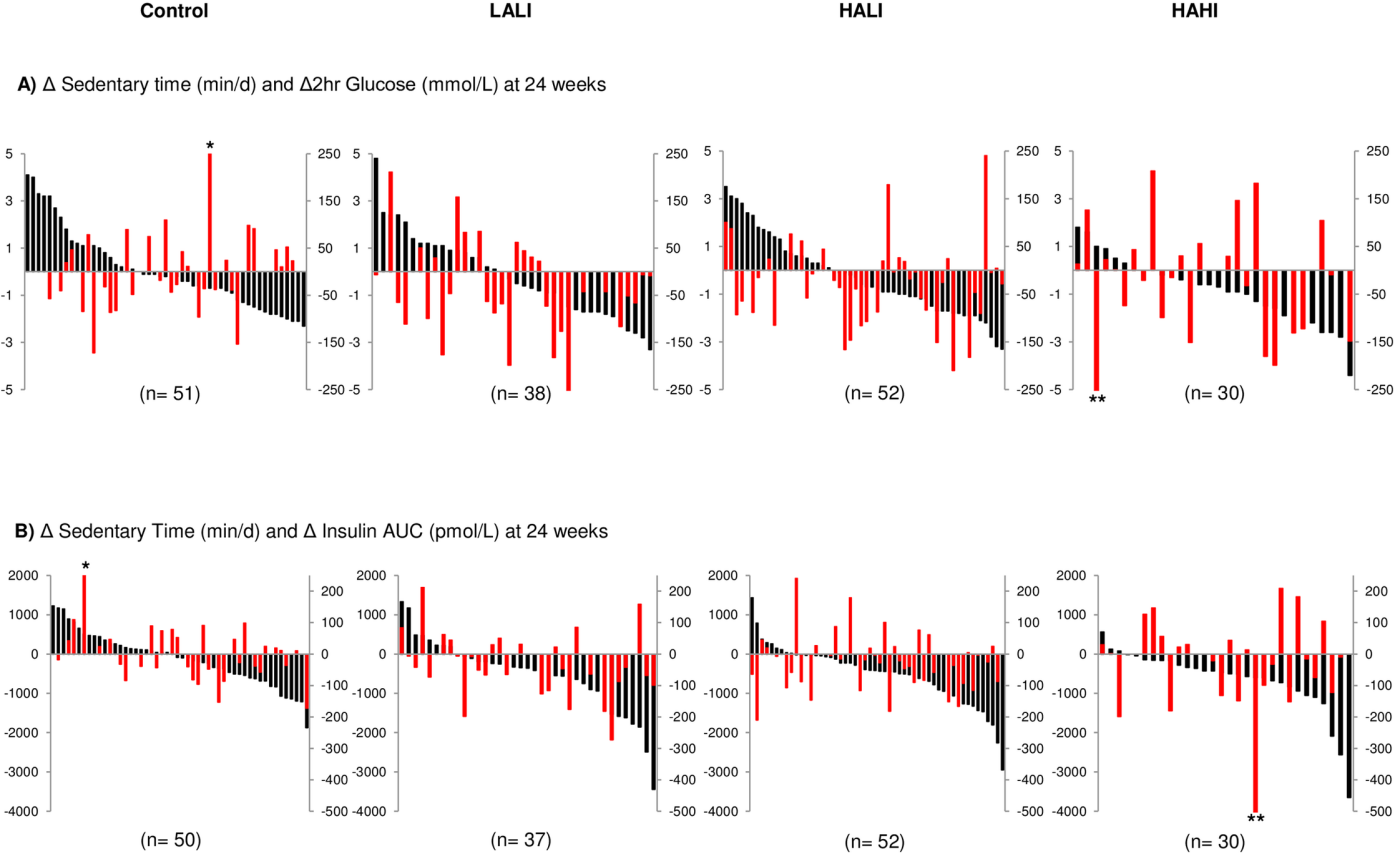
Change in sedentary time with corresponding change in 2-hour glucose and insulin AUC for each participant at 24 weeks across groups. (A) Change in sedentary time (red bars; min/d) with 2-hour glucose change (black bars; mmol/L) and (B) Change in sedentary time (red bars; min/d) with insulin AUC change (black bars; pmol/L). Data for fasting insulin not shown. LALI = low amount, low intensity exercise; HALI = high amount, low intensity exercise; HAHI = high amount, high intensity exercise. * indicates 377 min/d of sedentary time, ** indicates -598 min/d of sedentary time.

The individual change scores for sedentary time with 2-hr glucose and insulin AUC are illustrated in Fig 5. Change in total daily physical activity and sedentary time did not predict change in 2-hour glucose, insulin AUC, or fasting insulin within any group (p>0.05). There were no differences for change in total daily physical activity or sedentary time between responders and nonresponders for all variables within any group (p>0.05).

### Exercise adherence and rate of response

**Fig 6:**
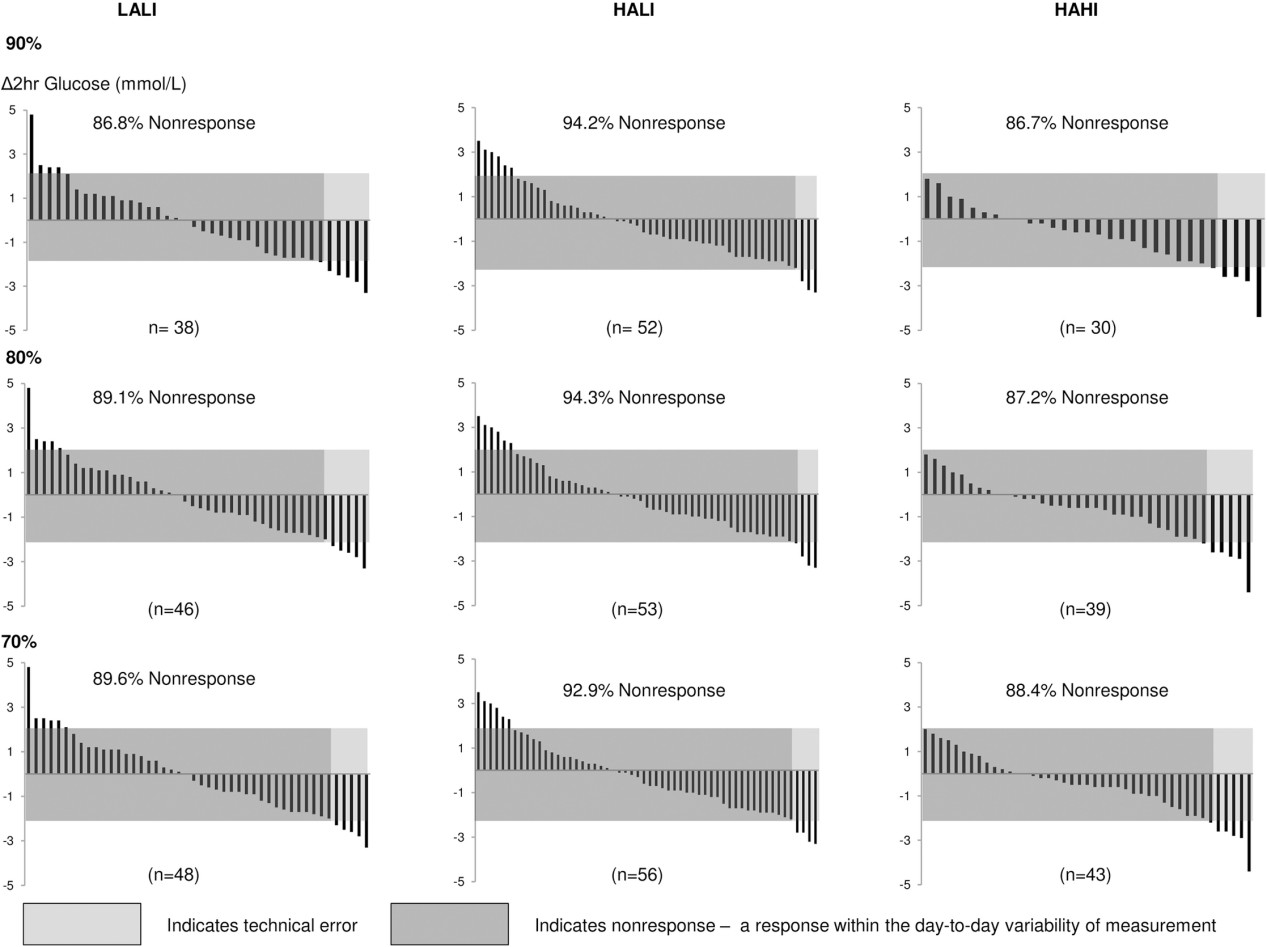
Change in 2-hour glucose for each participant at 24 weeks at 70%, 80%, and 90% adherence. LALI = low amount, low intensity exercise; HALI = high amount, low intensity exercise; HAHI = high amount, high intensity exercise.

**Fig 7:**
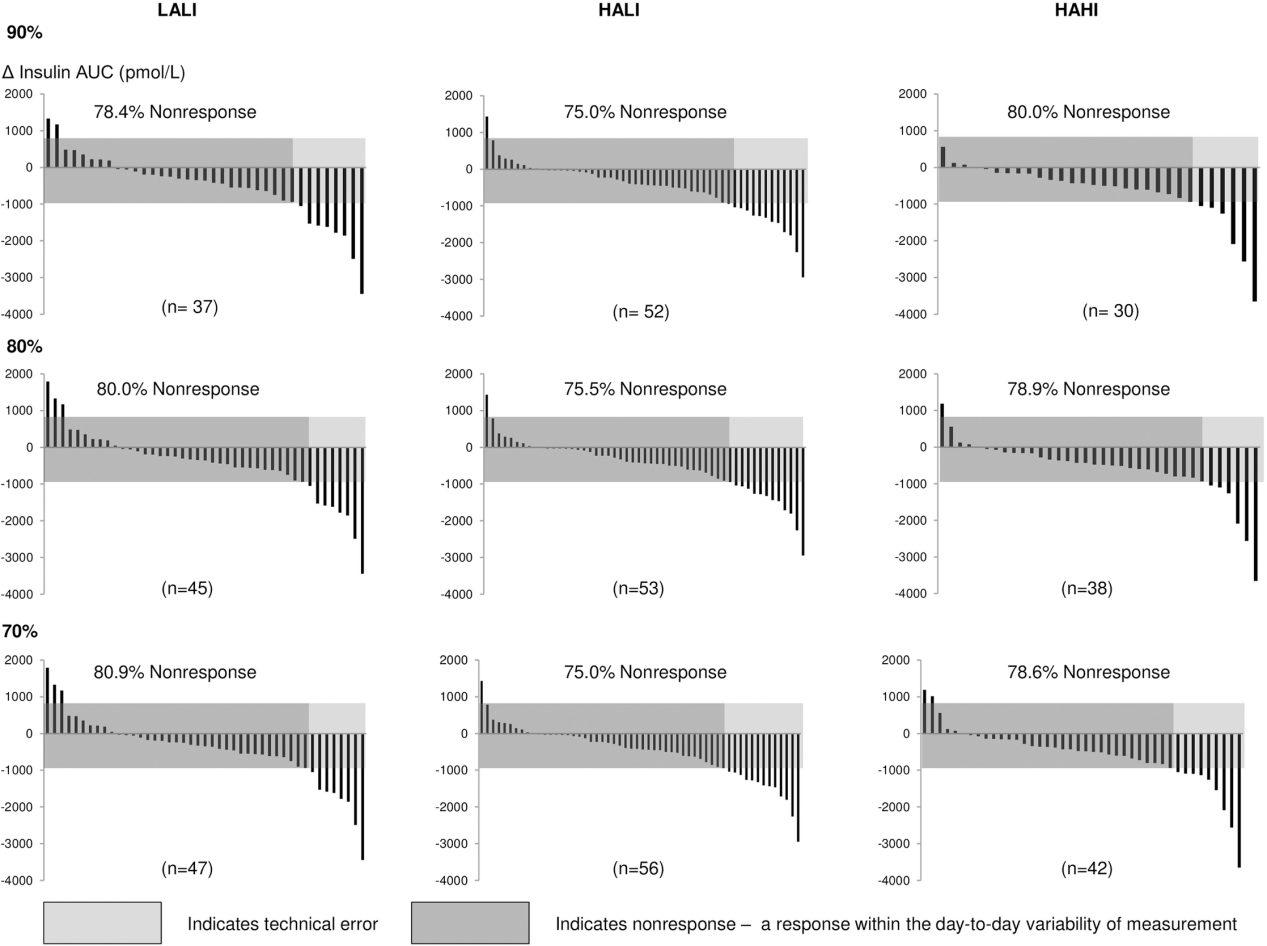
Change in insulin AUC for each participant at 24 weeks at 70%, 80%, and 90% adherence. LALI = low amount, low intensity exercise; HALI = high amount, low intensity exercise; HAHI = high amount, high intensity exercise.

**Fig 8:**
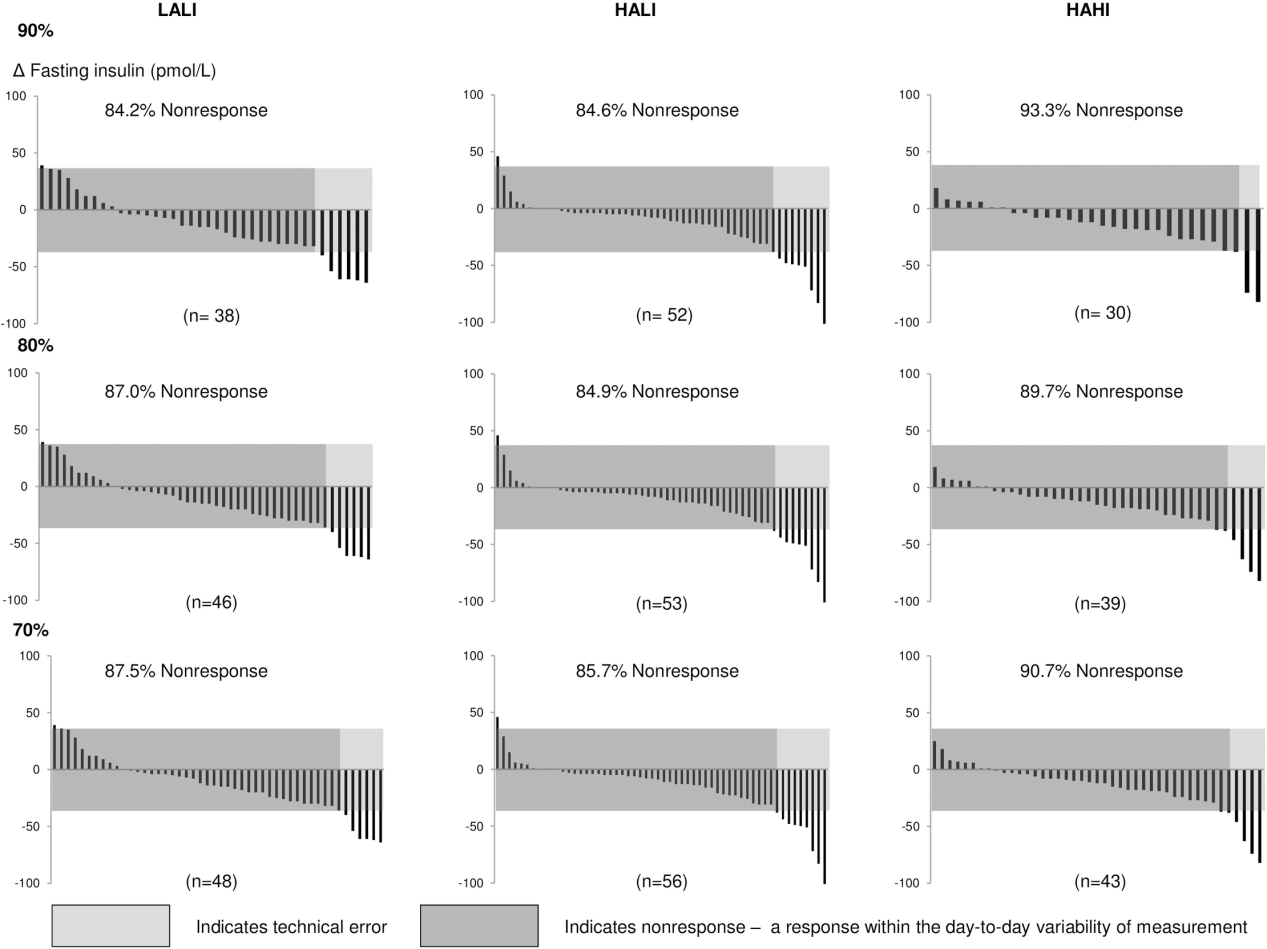
Change in fasting insulin for each participant at 24 weeks at 70%, 80%, and 90% adherence. LALI = low amount, low intensity exercise; HALI = high amount, low intensity exercise; HAHI = high amount, high intensity exercise.

Participants included in this analysis adhered to at least 90% (108 of 120) of the prescribed exercise sessions. Interestingly, for all variables, the pattern of response at lower levels of adherence (70% and 80%) was not materially different (Figs 6–8).

## Discussion

The primary finding of this ancillary study is that regardless of exercise amount or intensity, approximately 80% of the participants did not improve measures of glucose and insulin beyond the day-to-day variability of measurement. This observation underscores the substantial individual variability that occurs in response to standardized exercise, and the importance of accounting for the variability of measurement when interpreting treatment efficacy for a given individual.

Increasing physical activity combined with a balanced diet remains a cornerstone of diabetes prevention and treatment guidelines worldwide [21]. This recommendation derives from randomized trials and observational cohort studies the primary findings from which are based on the mean response of the cohort studied. Thus, practitioners who abide by the consensus recommendation approach the prevention and treatment of diabetes associated with physical inactivity based on the expected response from the average participant. Indeed, the findings from our primary report confirm that by comparison to control, the average decrease observed for 2-hr glucose was significant for HAHI, and that reduction in insulin AUC was significant for both HALI and HAHI [12]. However, the findings here provide strong evidence that the efficacy of exercise to improve glucose management may not apply to each member of that group; for example, insulin AUC ranged from approximately -3500 pmol/L to +1500 pmol/L in the LALI group. We have also shown that exercise amount (energy expenditure, kcal) or intensity (% of VO_2_max) do not appear to be major determinants of the individual variability in glucose or insulin response where the variability of response changed little when increasing either the exercise amount (approximately -3000 to +1500 pmol/L) or intensity (approximately -3500 to +500 pmol/L). That the individual variability in response to standardized exercise is not influenced by exercise amount or intensity extends the work of Bouchard and colleagues who have documented a substantial individual variability in response to regular exercise for several cardiometabolic variables [2, 22, 23].

We argue here that a true treatment benefit for a given individual is a response that exceeds the biological variability or our 2TE measurement. Though our 2TE values could be construed as conservative given that the vast majority of our participants did not exceed the variability of response observed within our control group, our 2TE values for insulin and glucose are consistent with those reported in the literature [2, 4, 7, 18, 19]. and are confirmed by the variability observed within our control group as the change scores for approximately 95% fell within 2 TEs. Had we used a change value greater than zero as the threshold for benefit, 50–90% of our participants would be characterized as responders to treatment. However, with this approach 50% of our control group participants would also be responders.

The heterogeneity of response observed in response to a rigorously controlled exercise regimen may not be surprising given the established variability in both intrinsic and acquired characteristics. Heritability estimates for fasting glucose range from 10 to 75% and fasting insulin from 20 to 55% [1]. In attempts to minimize the variability due to acquired characteristics we took care to track daily physical activity levels, sedentary time, and dietary composition of our participants throughout the intervention [11]. We required all participants to refrain from eating (12 hours), and exercise (24–48 hours) prior to measurement. If a participant violated these requirements the OGTT was rescheduled [11].

It is apparent from our findings, and others [2, 18, 24], that the day-to-day variability reported for insulin and glucose variables is extremely large. One method to reduce the variability of measurement is to take repeat measurements pre- and post-treatment [25]. Although obtaining multiple measures (e.g. 2–3) may present a burden to both participant and practitioner, the burden is offset by the confidence gained that an individual change value is beyond the day-to-day variability and hence, a true response to treatment.

Finally, we also explored whether exercise exposure influenced the rate of response. We previously reported that for cardiorespiratory fitness, the number of nonresponders to treatment plateaued at 16 weeks [8]. Similar to our findings with cardiorespiratory fitness, there was no further change in the rate of response in glucose and insulin measures between 16 and 24 weeks of exercise regardless of group suggesting that if individuals do not improve in measures of insulin and glucose by 16 weeks, it is unlikely that they will with further exposure to exercise.

Strengths of our study include rigorous control of exercise amount and intensity and accounting for the potential confounding effect of changes in daily physical activity and sedentary time. We included those individuals who adhered to at least 90% of the exercise program, which helped to ensure that the response for a given variable was not due to poor exercise attendance. Limitations include the observation that our study sample was primarily white and obese which may limit generalizability. We did not perform power calculations a-priori as we present findings from a secondary analysis. However, post-hoc analyses based on our findings suggest that to achieve 80% power to detect a 20% increase in rate of response between groups, 79 participants per arm or 316 participants in total would be required. Further, slightly less than 95% of the change scores for our control group did not fall within 2TEs, which may suggest that our 2TE value underestimated the number of nonresponders in our intervention groups, and that the number of true responders was less than observed. In addition, whether an individual response to one treatment would be altered in response to another is unknown. Finally, there are several factors that affect insulin and glucose variables that we did not control for such as: dietary composition of meals consumed the day before the OGTT [4], the degree of mastication [26], stress levels of the participants [6], sleep deprivation [5], and the phase of the menstrual cycle during testing [27].

In conclusion, our findings underscore the substantial individual variability that occurs in response to a standard exercise dose for glucose and insulin measures and the importance of accounting for the variability of measurement when interpreting treatment efficacy for a given individual.

## Supporting information

S1 File Dataset. Data used for the analysis performed in this paper.
(XLSX)
